# The combination of blue dye and radioisotope versus radioisotope alone during sentinel lymph node biopsy for breast cancer: a systematic review

**DOI:** 10.1186/s12885-016-2137-0

**Published:** 2016-02-16

**Authors:** Pei-Sheng He, Feng Li, Guan-Hua Li, Can Guo, Tian-Jin Chen

**Affiliations:** The Second Department of General Surgery, Chongqing Three Gorges Central Hospital, 165 Xincheng Road, Chongqing, 404000 China

**Keywords:** Breast cancer, Sentinel lymph node, Blue dye, Radioisotope, Systematic review

## Abstract

**Background:**

The combination of blue dye and radioisotope is most widely used to identify sentinel lymph nodes (SLNs) in patients with breast cancer. However, some individual studies suggested that dual tracers did not have an advantage over radioisotope alone in detecting SLNs. We performed a systematic review to investigate the added value of blue dye in addition to radioisotope.

**Methods:**

We searched Pubmed and Embase. Prospective studies that compared the combination of radioisotope and blue dye with radioisotope alone were selected. The identification rate of SLNs and the false-negative rate were the main outcomes of interest. The odds ratios (ORs) and 95 % confidential intervals (CIs) were calculated by using random-effects model.

**Results:**

Twenty-four studies were included. The combination of radioisotope and blue dye showed higher identification rate than radioisotope alone (OR = 2.03, 95 % CI 1.53–2.69, *P* < 0.05). However, no statistically significant difference was revealed for patients after neoadjuvant chemotherapy (OR = 1.64, 95 % CI 0.82–3.27, *P* > 0.05), or for studies with high proportion of patients with positive lymphoscintigraphy (OR = 1.41, 95 % CI 0.83–2.39, *P* > 0.05). Dual tracers did not significantly lower the false-negative rate compared with radioisotope alone (OR = 0.76, 95 % CI 0.44–1.29, *P* > 0.05).

**Conclusions:**

Although the combination of blue dye and radioisotope outperformed radioisotope alone in SLN detection, the superiority for dual tracers may be limited for patients with positive lymphoscintigraphy or for those after neoadjuvant chemotherapy. Besides, the combined modality did not help lower the false-negative rate.

## Background

The most important prognostic factor for patients with early-stage breast cancer was the disease status of axillary lymph nodes [1]. Recently, sentinel lymph node biopsy (SLNB) has replaced axillary lymph node dissection (ALND) to be the standard procedure for axillary staging in patients with clinically node-negative breast cancer [2, 3]. SLNs were defined as the first lymph nodes that received lymphatic drainage from the primary cancer. Since the early 1990s, blue dye and radioisotope have emerged as the most commonly used tracing agents to locate SLNs in breast cancer [4, 5]. In particular, the combined use of blue dye and radioisotope gained widespread popularity [6]. A previous survey of fellows of the American College of Surgeons showed that 90 % used the combined modality [7].

Notably, blue dye injection carried the potential risks of skin tattooing, skin necrosis, and allergic reactions [8]. Approximately 2 % of patients undergoing SLNB would experience allergic reactions to blue dye [9], with the most severe case presenting as hypotension [10]. Several authors argued that the added value of blue dye over radiotracer alone technique was only minimal or marginal [9–11]. The results from a large case series suggested that the marginal benefit for blue dye declined with increased surgical experience in radioisotope-mapping technique [12].

It is attractive to use radioisotope alone to avoid the blue-dye complications and lower the cost of hospital care. However, the current knowledge on the added value of blue dye is still based on weak evidence from scattered individual studies without universal consensus. A randomized controlled trial (RCT) has only recently been conducted to compare dual tracers with radioisotope alone in patients before neoadjuvant chemotherapy (NAC) with positive preoperative lymphoscintigraphy (LSG), which demonstrated no advantage for dual tracers in SLN detection [11]. The authors speculated that the blue dye should be added only for patients with negative LSG or those having received NAC. An evidence-based systematic review was warranted to identify patients who will particularly benefit from dual tracers, and to help inform SLNB decision-making. Thus, we conducted this systematic review regarding prospective studies on SLNB in breast cancer, aiming to gain a better understanding of the incremental value of blue dye in addition to radioisotope. Especially, the potential confounding clinical factors were explored.

## Methods

### Study selection

Electronic databases of Pubmed and Embase were systematically searched up to June 2015. The search terms used were: ‘sentinel lymph node’, ‘breast cancer’, ‘blue dye’ or ‘lymphazurin’ or ‘Isosulfan’ or ‘methylene blue’ or ‘patent blue’, ‘isotope’ or ‘radioisotope’ or ‘radiolabeled colloid’ or ‘radiocolloid’ or ‘radiotracer’. The search was restricted to human subjects and English language. All studies were critically appraised for inclusion eligibility. We also manually searched the reference lists of relevant studies.

### Inclusion criteria

Studies were considered for inclusion if they fulfilled the following criteria: (1) reported the use of blue dye and radioisotope for SNLB in female breast cancer patients; (2) showed the comparison between the combination of blue dye and radioisotope with radioisotope alone; (3) reported outcomes of the identification rate of SLNs or the false-negative rate; (4) prospectively collected patients’ data, designed as randomized controlled trial (RCT) or non-randomized prospective study (NPS); (5) enrolled at least 100 patients, with at least 20 patients available for each mapping strategy.

### Data extraction and quality assessment

Data from the included studies were extracted independently by two authors (PSH and GHL). Any discrepancy was resolved by consensus or by discussion with a third author (FL). The following information was extracted: author and publication year, location, study design, sample size, age, clinical status of axillary nodes, NAC use, mapping materials, injection site, and proportion of patients with positive preoperative LSG. The identification rate of SLNs and the false-negative rate were directly extracted or indirectly calculated for each mapping strategy. The quality of studies were appraised by a revised 6-item scale which was derived from a previous 5-item scale [13]. Assuming that the success rate of SLN identification reached the level of 98 % for dual mapping agents, and differed by 5 % between dual and single tracing agents, accompanied by a Type I error probability for a two-sided test of 5 % and statistical power of 80 %, the required sample size in each group was calculated to be approximately 300 [14]. Thus, we listed the sufficiency of sample size as one item on the quality scale. The quality assessment included the following elements: 1) describing patients’ characteristics, 2) explaining reasons for withdrawal, 3) describing measures of outcomes, 4) incorporating measures of confounding factors, 5) describing the SLN technique (mapping material and injection site), and 6) enrolling at least 300 patients. The study with 5 points or more was regarded as high quality.

### Statistical analysis

The odds ratios (ORs) and 95 % confidential intervals (CIs) were used as statistical measures for dichotomous outcomes. They were calculated from the number of patients in each mapping modality. The identification rate of SLNs and the false-negative rate were considered as the main outcomes. The random-effects model was used to calculate the summary effect estimates [15]. The heterogeneity between studies was analyzed by the I^2^ statistics and Cochrane Q test, with I^2^ > 50 % and *P* < 0.05 deemed as significant heterogeneity. The source of heterogeneity was explored by subgroup analysis, meta-regression and cumulative analysis. The following predefined covariates were considered into subgroup analyses: clinical node status (negative or positive), NAC use (before NAC or after NAC), proportion of patients with positive preoperative LSG (≥90 % or < 90 %), sample size (>300 or <300), data source (RCT or NPS), and injection site of mapping materials (superficial or deep). The superficial injection sites included periareolar, subareolar, intradermal, and subdermal; the deep injection sites included peritumoral, intratumoral, and intraparenchymal [16]. Meta-regression analysis was performed according to the sample size, publication year, and the proportion of patients with positive preoperative LSG. The cumulative analysis was conducted according to the publication year. The publication bias was examined visually by the funnel plot and statistically by the Egger’s test. *P* < 0.05 was considered to represent statistically significance. The statistical analyses were performed by the STATA 12.0 (StataCorp LP, College Station, Texas, USA).

Additionally, we pooled the false-negative rates and the incidence of adverse reactions caused mapping agents, which were processed by the software of Comprehensive Meta-Analysis statistical package (CMA Version 2.2, Biostat, Englewood, NJ), with the use of random-effects model.

## Results

### Literature search

**Fig. 1:**
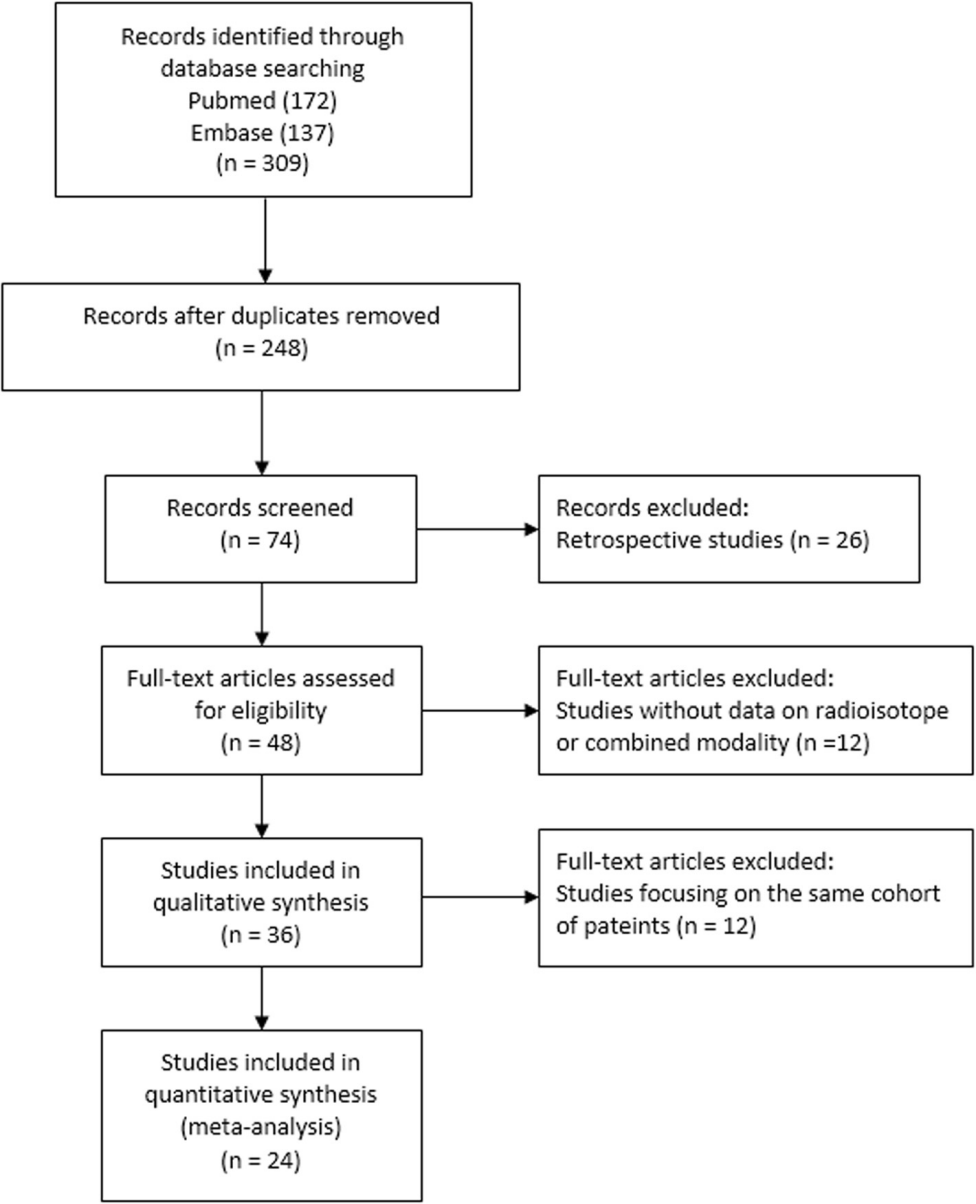
The flow diagram of literature search process

A total of 309 citations were identified after the initial search, including 137 citations from Embase and 172 citations from Pubmed. Sixty-one duplicated records were excluded. Then we excluded reviews, case reports, editorials, studies with small sample sizes (<100), and studies of irrelevant topics. Seventy-four studies were screened by titles and abstracts. After excluding 26 retrospective studies, the full-texts of 48 records were assessed for eligibility. Data on the combined mapping modality could not be obtained from 12 studies. Twelve studies enrolled patients of the duplicated cohorts. Finally, twenty-four studies were selected for meta-analyses [1, 3, 8, 10–12, 17–34]. The flow diagram of selection process was depicted in Fig. 1.

### Study characteristics and quality assessment

A total of 15,462 patients with breast cancer undergoing SLNB were involved. The characteristics of 24 included studies were presented in Table 1. The sample size ranged from 100 to 3402, with 15 studies of sample sizes over 300 and 9 studies of sample sizes below 300. Eight studies were conducted in the United States, 11 in Europe, and 5 in Asia. Six studies were designed as RCTs. However, only one RCT was primarily designed to compare radioisotope alone with the combined use of radioisotope and blue dye [11]. The comparison data were retrieved from post-hoc analyses for other five RCTs. Eighteen studies were non-randomized prospective studies. The study qualities were appraised by the revised 6-item scale. The overall assessment was satisfactory with all scores ranging from 3 to 6. Most studies clearly described the patients’ characteristics (21/24), the details of SLN procedures (20/24), the confounding factors (21/24), and the measures of outcomes (24/24). However, the explanation of withdrawal or the selection process of participants were clearly stated by only few studies (7/24). The quality assessment was shown in Table 2.

**Table 1:** Characteristics of included studies

Author (year)	Design	Location	Sample size	Age	Clinical node status	NAC use	Radioisotope type	Blue dye type	Site of Blue dye	Site of isotope	Positive Preo LSG, No. (%)
Bass et al. (1999) [18]	NPS	USA	700	58	Unknown	Unknown	Filtered ^99m^Tc-sulfur colloid	Isosulfan	Intraparenchymal	Intraparenchymal	Unknown
Mariani et al. (2000) [28]	NPS	Italy	284	59	Mixed	Unknown	^99m^Tc-human albumin	Patent blue	Subdermal	Subdermal	Unknown
Rahusen et al. (2000) [30]	NPS	Netherlands	115	54	Unknown	Unknown	^99m^Tc-human albumin	Patent blue	intradermal	Intraparenchymal	105/115 (91 %)
Derossis et al. (2001) [12]	NPS	USA	2000	Unknown	Negative	Unknown	Unfiltered ^99m^Tc-sulfur colloid	Isosulfan	Intraparenchymal	Intradermal	Unknown
Bauer et al. (2002) [19]	NPS	USA	332	55	Negative	Unknown	Filtered ^99m^Tc-sulfur colloid	Isosulfan	Subareolar versus peritumoral	Peritumoral	195/223 (87.4 %)
Ahrendt et al. (2002) [17]	NPS	USA	174	59	Negative	Unknown	Filtered ^99m^Tc-sulfur colloid	Isosulfan	Intraparenchymal	Intraparenchymal	Unknown
Tsunoda et al. (2002) [34]	NPS	Japan	376	Unknown	Mixed	No	Tin colloid or phytate	Unknown	Subareolar or peritumoral	Peritumoral	Unknown
Pelosi et al. (2003) [29]	NPS	Italy	150	62	NA	Unknown	^99m^Tc-labelled Nanocoll	Isosulfan	Periareolar or subdermal	Periareolar or subdermal	93/100 (93 %)
Fleming et al. (2003) [22]	NPS	Ireland	125	≈56	Negative	Unknown	Radiocolloid isotope	Isosulfan	Periareolar	Intraparenchymal versus intradermal	103/125 (82.4 %)
Schirrmeister et al. (2004) [32]	NPS	Germany	814	58	62.9 % negative	Unknown	Radioactive colloid	Isosulfan or patent blue	Optional	Optional	Unknown
Lauridsen et al. (2004) [24]	NPS	Denmark	124	56	Negative	Unknown	^99m^Tc-human albumin	Patent blue	Peritumoral	Peritumoral	Unknown
Mamounas et al. (2005) [27]	RCT	USA	428	Unknown	76.2 % negative	Yes	Unknown	Isosulfan	Unknown	Unknown	Unknown
Takei et al. (2006) [33]	NPS	Japan	308	55	Negative	Unknown	^99m^Tc-phytate	Patent blue	Subdermal	Subdermal	Unknown
Argon et al. (2006) [1]	NPS	Turkey	100	48	Negative	No	^99m^Tc-tin colloid	Isosulfan	Intraparenchymal	Intradermal	90/100 (90 %)
Low et al. (2006) [26]	NPS	Australia	113	56	Negative	Unknown	^99m^Tc-sulfur colloid	Patent blue	Intradermal or subdermal	Peritumoral	97/113 (85.8 %)
Goyal et al. (2006) [23]	RCT	UK	842	18–80	Negative	Unknown	^99m^Tc-albumin colloid	Patent blue	Peritumoral	Peritumoral	490/707 (69.3 %)
Lelievre et al. (2007) [25]	NPS	France	152	57	Unknown	Unknown	^99m^Tc-sulfur colloid	Patent blue	Subareolar or peritumoral	Intradermal and intraparenchymal	149/152 (98 %)
Rodier et al. (2007) [31]	RCT	France	449	25–90	Negative	No	^99m^Tc-sulfur colloid	Patent blue	Peritumoral versus periareolar	Peritumoral versus periareolar	353/432 (81.7 %)
Kang et al. (2010) [10]	NPS	USA	3402	56	Negative	Mixed	^99m^Tc-sulfur colloid	Isosulfan	Unknown	Unknown	1566/1720 (91.0 %)
Johnson et al. (2011) [8]	NPS	USA	696	57	Unknown	Unknown	Unfiltered ^99m^Tc- sulfur colloid	Isosulfan	Subareolar	Subareolar	Unknown
Kuehn et al. (2013) [3]	NPS	Germany, Austria	1334	49	Negative	Mixed	Unknown	Unknown	Optional	Optional	1490/1614 (92.3 %)
Boughey et al. (2013) [20]	RCT	USA	689	49 (23–93)	Positive	Yes	Unknown	Isosulfan or methylene	Optional	Optional	Unknown
Elmadahm et al. (2015) [21]	RCT	Australia	1088	Unknown	Negative	No	^99m^Tc-sulfur colloid	Patent blue	Peritumoural	Peritumoural	779/957 (81.4 %)
O'Reilly et al. (2015) [11]	RCT	Ireland	667	48	Negative	No	Unknown	Isosulfan	Intradermal	Subdermal	667/667 (100 %)

*FNR* false-negative rate, *LSG* lymphoscintigraphy, *NAC* neoadjuvant chemotherapy, *NPS* non-randomized prospective study

**Table 2:** Quality assessment of included studies by a revised 6-item scale

Author (year)	Description of patients’ characteristics	Reasons for withdrawal	Description of measures of outcomes	Evaluation of confounding factors	Description of the SLN technique	Sample size over 300	Total score
Bass et al. (1999) [18]	0	0	1	1	1	1	4
Mariani et al. (2000) [28]	1	0	1	0	1	0	3
Rahusen et al. (2000) [30]	1	0	1	0	1	0	3
Derossis et al. (2001) [12]	1	0	1	1	1	1	5
Bauer et al. (2002) [19]	1	0	1	1	1	1	5
Pelosi et al. (2003) [29]	1	0	1	1	1	0	4
Fleming et al. (2003) [22]	1	0	1	1	1	0	4
Ahrendt et al. (2002) [17]	1	0	1	1	1	0	4
Tsunoda et al. (2002) [34]	0	0	1	1	1	1	4
Schirrmeister et al. (2004) [32]	1	0	1	0	0	1	3
Lauridsen et al. (2004) [24]	1	1	1	1	1	0	5
Mamounas et al. (2005) [27]	1	1	1	1	0	1	5
Takei et al. (2006) [33]	1	0	1	1	1	1	5
Argon et al. (2006) [1]	1	0	1	1	1	0	4
Low et al. (2006) [26]	1	1	1	1	1	0	5
Goyal et al. (2006) [23]	1	0	1	1	1	1	5
Lelievre et al. (2007) [25]	1	0	1	1	1	0	4
Rodier et al. (2007) [31]	1	1	1	1	1	1	6
Kang et al. (2010) [10]	1	0	1	1	0	1	4
Johnson et al. (2011) [8]	0	0	1	1	1	1	4
Kuehn et al. (2013) [3]	1	1	1	1	0	1	5
Elmadahm et al. (2015) [21]	1	0	1	1	1	1	5
O'Reilly et al. (2015) [11]	1	1	1	1	1	1	6
Boughey et al. (2013) [20]	1	1	1	1	1	1	6

### Identification rate of SLNs

**Fig. 2:**
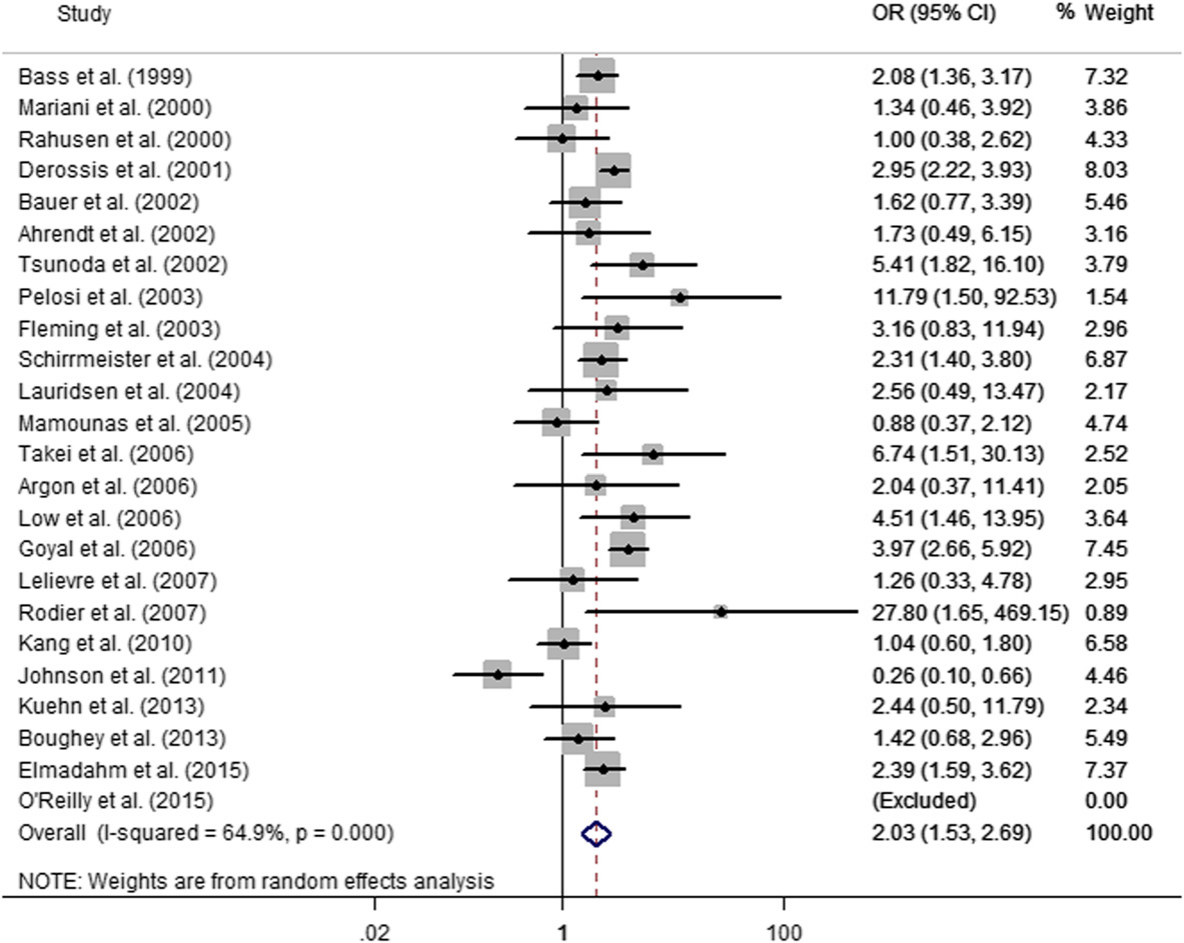
Forest plot showing that the combined use of blue dye and radioisotope showed higher SLN identification rate than radioisotope alone

All of the 24 studies compared the the identification rate of SLNs between dual tracers and radioisotope alone. The pooled results demonstrated that the combined use of radioisotope and blue dye had higher identification rate of SLNs than radioisotope alone (OR = 2.03, 95 % CI 1.53–2.69, *P* < 0.05) (Fig. 2). Significant heterogeneity was detected (I^2^ = 64.9 %, *P* < 0.05). The primary subgroup analyses were conducted according to the clinical node status, NAC use (before NAC or after NAC), and proportion of patients with positive preoperative LSG (≥90 % or < 90 %).

### The impact of clinical node status

The clinical axillary node status was exclusively negative in 14 studies, exclusively positive in 1 study, mixed in 4 studies, and not clear in 4 studies (Table 1). In the subgroup of 14 studies with clinically node-negative breast cancer, the pooled data indicated that the use of dual tracers was superior to radioisotope alone in identifying SLNs (OR = 2.56, 95 % CI 1.88–3.49, *P* < 0.05; I^2^ = 48.7 %). However, no significant results were revealed for other subgroups (Table 3).

**Table 3:** Subgroup analyses of studies on the sentinel lymph node identification

Subgroups	No. of studies	OR	95 % CI	*P* value	Heterogeneity (I^2^)
Clinical node status					
Negative	14	2.56	1.88–3.49	<0.05	48.7
Positive	1	1.42	0.68–2.96	>0.05	–
Mixed	4	1.93	0.99–3.76	>0.05	59.5
NAC					
Before NAC	6	2.96	1.78–4.94	<0.05	15.6
After NAC	3	1.53	0.94–2.47	>0.05	31.6
Proportion of patients with positive LSG					
≥ 90 %	7	1.41	0.83–2.39	>0.05	22.1
< 90 %	6	2.99	1.99–4.48	<0.05	42.8
Data source					
RCT	6	2.25	1.23–4.11	<0.05	74.7 %
NPS	18	1.96	1.40–2.74	<0.05	62.5 %
Sample size					
> 300	15	2.01	1.42–2.84	<0.05	75.9 %
< 300	9	2.01	1.28–3.15	<0.05	7.0 %
Location					
USA	8	1.33	0.82–2.16	>0.05	80.1 %
Europe	11	2.48	1.60–3.84	<0.05	43.7 %
Asia	5	2.93	2.05–4.19	<0.05	1.3 %
Injection site of blue dye					
Superficial	10	1.95	0.93–4.08	>0.05	70.6 %
Deep	9	2.76	2.32–3.30	<0.05	0
Injection site of radioisotope					
Superficial	9	2.05	0.87–4.84	>0.05	76.0 %
Deep	11	2.55	1.93–3.37	<0.05	31.2 %

*LSG* lymphoscintigraphy, *NAC* neoadjuvant chemotherapy

### The impact of neoadjuvant chemotherapy

The use of NAC was clearly described by 8 studies, including 5 studies of patients before NAC, 2 studies of patients after NAC, and 2 studies with mixed populations (Table 1). Kuehn et al. reported both data for patients before NAC and those after NAC, which were extracted separately [3]. For 6 studies including patients before NAC [1, 3, 11, 21, 31, 34], the combined use of blue dye and radioisotope showed higher identification rate than radioisotope alone (OR = 2.96, 95 % CI 1.78–4.94, *P* < 0.05; I^2^ = 15.6 %). For 3 studies including patients after NAC [3, 20, 27], no statistically significant difference was revealed when comparing dual tracers with radioisotope alone (OR = 1.53, 95 % CI 0.94–2.47, *P* > 0.05; I^2^ = 31.6 %) (Table 3).

### The impact of preoperative LSG

The proportion of patients with positive preoperative LSG was reported by 13 studies, ranging from 69.3 to 100 % (Table 1). For 7 studies with a high proportion (≥90 %) [1, 3, 10, 11, 25, 29, 30], the pooled data revealed no statistically significant difference between dual tracers and radioisotope alone (OR = 1.41, 95 % CI 0.83–2.39, *P* > 0.05). For 6 studies with a relatively low proportion (<90 %) [19, 21–23, 26, 31], the advantage of using dual tracers was statistically significant (OR = 2.99, 95 % CI 1.99–4.48, *P* > 0.05) (Table 3).

### Stratified analyses

Additionally, subgroup analyses were conducted according to the data source (RCT or NPS), sample size (over 300 or below 300), location (USA, Europe, or Asia), injection site of blue dye (superficial or deep), and injection site of radioisotope (superficial or deep). Three studies used both superficial injection and deep injection [19, 22, 31]. Related data were extracted separately. The results remained significant in most subgroup analyses. However, no statistically significant difference was shown between dual tracers and radioisotope alone for patients receiving superficial injection of blue dye (OR = 1.95, 95 % CI 0.93–4.08, *P* > 0.05), or for those receiving superficial injection of radioisotope (OR = 2.05, 95 % CI 0.87–4.84, *P* > 0.05). Results for subgroup analyses were summarized in Table 3.

### Meta-regression and cumulative analysis

**Fig. 3:**
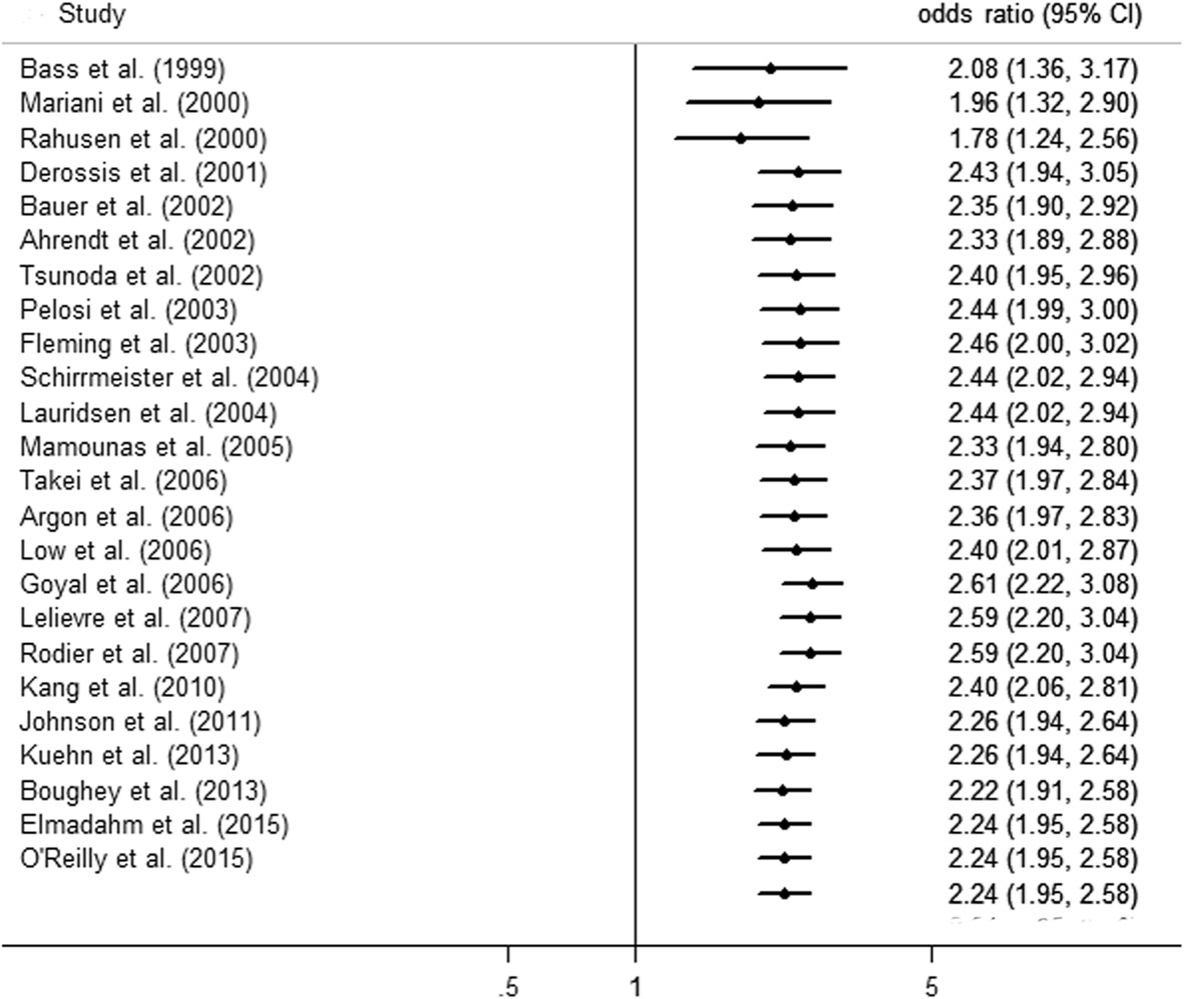
Cumulative meta-analysis according to the publication year showing that the advantage of dual tracers remained stable over years

The publication year and sample size were considered as independent variables into meta-regression analyses. No significant independent effect was detected for publication year (*P* = 0.37) or sample size (*P* = 0.52). Meta-regression was also performed for 13 studies reporting the proportion of patients with preoperative LSG, which showed a significant independent effect of this covariate (*P* < 0.01). Assumed that the surgical experience in mapping techniques increased over years, cumulative analysis was performed to investigate the effect of publication year. Notably, the advantage of combined mapping modality was stable over years (Fig. 3).

### Publication bias

**Fig. 4:**
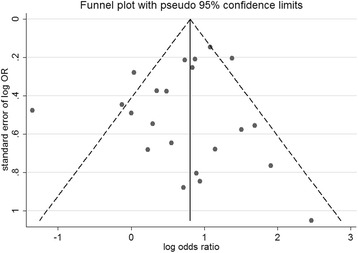
Funnel plot showing that no evidence of publication bias was identified

The funnel plot was visually symmetrical (Fig. 4). No statistical significance was detected by Egger’s test (*P* = 0.34).

### False-negative rate

**Fig. 5:**
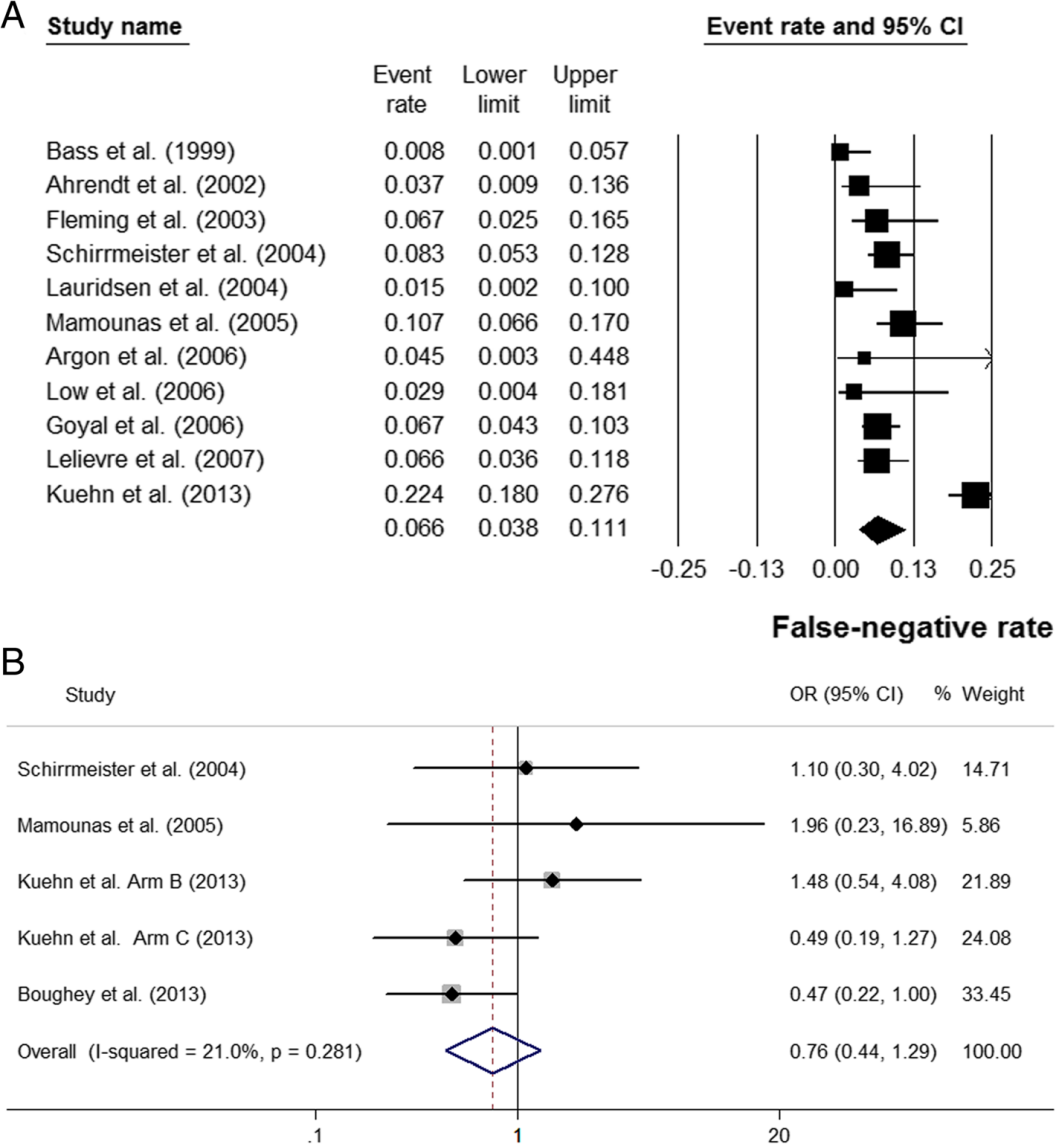
Forest plots showing the pooled false-negative rate, and the comparison between dual tracers and radioisotope alone in false-negative rate. **a** pooled false-negative rate **b** Forest plot of ORs showing that the combination of blue dye and radioisotope did not significantly decrease the false-negative rate when compared with radioisotope alone

The false-negative rate was investigated by 12 studies [1, 3, 17, 18, 20, 22–27, 32]. The pooled false-negative rate was 7.5 % (95 % CI 4.8–11.5 %), with significant heterogeneity (I^2^ = 82.4 %, *P* < 0.05) (Fig. 5a). However, only 4 studies reported the comparison of false-negative rate between radioisotope alone and the combined method [3, 20, 27, 32]. Kuehn et al. reported the false-negative rate in two subgroups, and they were separately analyzed [3]. The combined use of radioisotope and blue dye did not significantly lower the false-negative rate when compared with radioisotope alone (OR =0.76, 95 % CI 0.44–1.29, *P* > 0.05). No significant heterogeneity was detected (I^2^ = 21.0 %, *P* > 0.05) (Fig. 5b).

### Adverse reactions

**Fig. 6:**
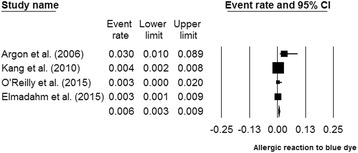
Forest plot showing the pooled incidence of allergic reaction to blue dye

Of the 24 publications, no study reported adverse episodes for the use of radioisotope. In contrast, 4 studies reported allergic reactions to blue dye [1, 10, 11, 21]. Most patients experienced mild allergic reactions. However, Kang et al. reported 5 cases of serious allergic reactions presenting as hypotension among 2049 patients [10]. The pooled incidence of allergic reaction to blue dye was 0.6 % (95 % CI 0.2–1.7 %), with significant heterogeneity (I^2^ = 72.5 %, *P* < 0.05) (Fig. 6).

## Discussion

This systematic review included 24 studies involving 15,462 participants. To our knowledge, it represented the largest and most comprehensive systematic review of prospective studies investigating the added value of blue dye in addition to radioisotope alone for tracing SLNs in breast cancer. It reflected the worldwide experience over 20 years. The overall pooled analysis showed that the combination of radioisotope and blue dye was superior to radioisotope alone for the successful identification of SLNs. The pooled false-negative rate was 7.5 %, which was similar to the pooled rate of 7.3 % in a previous meta-analysis [13]. Notably, the combined use of blue dye and radioisotope failed to confer significant advantage in lowering the false-negative rate.

The recent RCT failed to demonstrate an advantage with the addition of blue dye to radioisotope alone in patients before NAC with positive preoperative LSG [11]. LSG is a useful tool to establish abnormal lymphatic drainage patterns, and to detect extra-axillary nodes particularly internal mammary nodes [21, 24, 30]. It is an integral part of single radioisotope tracer during SLNB. Half of our included studies have performed preoperative LSG. When pooling results for studies enrolling over 90 % patients with positive LSG, no advantage was demonstrated for using dual tracers. Meta-regression analysis revealed that preoperative LSG appeared to be the source of heterogeneity. These results were consistent with the recent trial that positive preoperative LSG may preclude the additional use of blue dye [11]. Several studies have suggested that the uptake of radioisotope was less favorable after NAC compared with primary surgery, which may decrease the detection rate of SLNs [3, 35, 36]. Nevertheless, we failed to show the advantage of dual tracers for patients after NAC. Additionally, the statistical significance was not shown in the subgroups of superficial injection of blue dye or radioisotope. For a long time, the optimal injection sites of mapping agents were controversial. A previous meta-analysis suggested that both superficial and deep injections of radioisotope and blue dye were effective for identifying SLNs, but failing to show statistical difference between the two methods [16]. Interestingly, in 3 studies comparing superficial injection with deep injection, the blue and hot concordance was higher in the superficial-injection group compared with the deep-injection group (90 % versus 87 %, 95.5 % versus 85.5 %, and 95.6 % versus 91.5 %, respectively) [19, 22, 31]. Thus, we inferred that the high concordance of blue dye and radioisotope for superficial injection may weaken the additional value of blue dye.

The learning curve was associated with the successful rate of SLN identification. It was estimated that 23 patients were required by an individual surgeon to achieve a 90 % ± 4.5 % success rate and 53 patients were required to achieve a 95 % ± 2.3 % success rate [18]. The marginal benefit of blue dye was shown to be significant during the initial learning period, but declining with increased experience in using radioisotope alone [10, 12]. Assuming that breast surgeons have gained more experience in mapping techniques during the recent years, cumulative and meta-regression analyses were conducted according to the publication year. However, no statistical significance was detected.

One major concern for blue dye was the potential risk of complications, which were infrequent but significant, including anaphylaxis, skin tattooing, and skin necrosis at the injection site [8]. Our results showed that the incidence of allergic reaction to blue dye was at a low level of 0.6 %. The allergic reactions to blue dye were categorized into grade 1 (urticaria or blue hives, pruritis, or a generalized rash), grade 2 (hypotension not requiring vasopressors), or grade 3 (hypotension requiring pressor support) [37]. Most allergic episodes in our included studies were of grade 1, and only Kang et al. reported the hypotension episodes of grade 2 (5/2049) and grade 3 (2/2049) [10]. In addition, blue dye may be unavailable in some institutions due to nationwide shortage. The localization of SLNs was surgeon-dependent for mapping with blue dye [18]. Although the cost of methylene blue was low, the charge for lymphazurin reached as high as approximately $600 in the USA [8]. Thus, the conversion from dual tracers to a single radioisotope injection would help facilitate the biopsy procedure, reduce complications, and diminished cost as well as resource utilization.

Several limitations of our study should be acknowledged. The number of included studies was small for the outcome of false-negative rate. Only a minority of the selected studies recorded the events of allergic reactions to blue dye in follow-up. Especially, only 1 RCT was primarily designed to compare dual tracers with radioisotope alone [11]. Most studies were non-randomized studies or post-hoc analyses of RCTs. The reason for choice of tracer was unknown for most studies. Thus, the selection bias may exist. It was difficult to match the age, race, distribution of clinical stages, relation to NAC, type of radiotracer, injection site of mapping agents, and surgeons’ experience between the comparison groups. These confounding factors may affect the identification rate as well as the false-negative rate. Skip metastasis, intraoperative pathological technique, and lymphatic vessel obstruction have been suggested to the main reasons for false-negative results [38], which may overweigh the influence of mapping-agents choice.

## Conclusion

In conclusion, compared with radioisotope alone, blue dye plus radioisotope showed a higher success rate of SLN identification. Nevertheless, the added value of blue dye appeared to be limited for patients with positive preoperative LSG, having received NAC, or undergoing superficial injection of tracing agents. Dual tracers were unhelpful for lowering the false-negative rate of SLNs. Considering the adverse reactions and inconvenience caused by blue dye injection, and the increased experience in using radioisotope, the advantage of dual tracers over radioisotope alone may be overestimated. Further well-designed randomized studies are required to recognize the sub-population in whom the dual tracers is especially required.
